# Controversies on the Use of Ultraviolet Rays for Disinfection During the COVID-19 Pandemic

**DOI:** 10.21315/mjms2021.28.1.15

**Published:** 2021-02-24

**Authors:** Chii Chii Chew, Philip Rajan

**Affiliations:** 1 Clinical Research Centre, Hospital Raja Permaisuri Bainun, Ministry of Health Malaysia Level 4, Ambulatory Care Centre (ACC), Ipoh, Perak, Malaysia; 2 School of Pharmaceutical Sciences, Universiti Sains Malaysia, Pulau Pinang, Malaysia; 3 Department of Ear, Nose and Throat (ENT), Hospital Raja Permaisuri Bainun, Ministry of Health Malaysia, Ipoh, Perak, Malaysia

**Keywords:** ultraviolet rays, SARS-Cov-2, COVID-19, disinfection, virus inactivation

## Abstract

During the coronavirus disease 2019 (COVID-19) pandemic, the use of ultraviolet (UV) rays to disinfect skin areas, clothes and other objects at the entry/exit points of public spaces has been widely discussed by stakeholders. While ultraviolet germicidal irradiation (UVGI) has been shown to effectively inactivate coronaviruses, including severe acute respiratory syndrome coronavirus (SARS-CoV)-1 and Middle East respiratory syndrome coronavirus (MERS-CoV), no specific evidence proves that it effectively inactivates the new SARS-CoV-2 virus that causes COVID-19. Because UV rays damage human tissue, UVGI should be used with caution and not directly on human skin. Various guidelines recommend that UVGI should not be used as a sole agent for disinfecting surfaces or objects but as an adjunct to the latest standard disinfecting procedures.

## Introduction

It has been increasingly suggested that ultraviolet (UV) tunnels or rays be used at building entrance points to decontaminate skin and objects during the coronavirus disease 2019 (COVID-19) outbreak. In scientific, medical and technical contexts, UV rays [also known as ultraviolet germicidal irradiation (UVGI)] are established as a disinfection measure. They are widely used to disinfect surfaces and objects in hospitals and food processing facilities. Historically, UVGI involved ultraviolet-C (UV-C), a short wavelength (100 nm–280 nm) radiation that inactivates microbes in air or water and on surfaces (1). The current preferred UVGI method for disinfecting emerging viral pathogens that cause epidemic or pandemic infectious diseases, however, uses gamma irradiation. The use of cobalt-60 as a source of gamma irradiation is well established for disinfection within the biocontainment field (2).

UV irradiation is germicidal mainly because its wavelength corresponds to the peak UV absorption of protein, ribonucleic acid (RNA) and deoxyribonucleic acid (DNA). UV absorption into pathogen genomes triggers radiolytic cleavage or radical reactions, causing changes in the pathogen’s nucleic acids that eventually lead to inactivation (2, 3). UV rays that inactivate double-stranded DNA viruses are thought to also inactivate single-stranded RNA genomes such as coronaviruses (4, 5). UV irradiation has proved effective as an adjunct disinfection process by inactivating severe acute respiratory syndrome coronavirus (SARS-CoV)-1 and Middle East respiratory syndrome coronavirus (MERS-CoV), the coronaviruses that cause severe acute respiratory syndrome (SARS) and Middle East respiratory syndrome (MERS), respectively (6).

## Controversial Use of UV Irradiation for Disinfection

While there is no specific vaccine or treatment for the new SARS-CoV-2 virus that causes COVID-19, the use of UV irradiation for disinfection, especially in public areas or on human skin, has been suggested. To date, however, the evidence that gamma irradiation inactivates viruses remains limited. A single study has shown a target dose of 20 kGy (2 MRad) gamma irradiation being absorbed by enveloped viruses (including coronaviruses) and resulting in inactivation. Absorption of gamma irradiation is affected by two main factors: irradiation conditions (e.g. temperature and distance from irradiation source) and qualities of the sample to be disinfected (e.g. origin, composition and volume) (2).

Theoretically, as part of the coronavirus family, the SARS-CoV-2 virus, which has a diameter of approximately 60 nm–140 nm, should be susceptible to UV inactivation (7), but there is no specific evidence for this (8). While there is no solid evidence that UVGI inactivates SARS-CoV-2, some authors suggest its use as an additional disinfection following a manual chemical disinfection process (8). Similarly, the Food and Drug Administration (FDA) suggests using UVGI to augment disinfection of healthcare environments after manual cleaning (9).

The FDA guidelines specify that while UVGI reduces the number of pathogens, it does not eliminate the virus completely. The practice should therefore be used as an adjunct to establish disinfection practices, not as a replacement. The time, distance and maximum exposure area should be considered when using UVGI, as the rays can be blocked by dirt or other obstructions and shadowed areas will not be disinfected. The main concern about unrestricted public use of UV devices, however, especially for sterilising skin, is that UV exposure may increase the risk of skin cancer (9).

The unverified effectiveness of using UV disinfection lamps to kill the SARS-CoV-2 virus was addressed by the World Health Organization (WHO), which emphasised that UV lamps should not be used to sterilise skin because they can cause skin irritation (10). This warning implies that UV-emitting devices should not be installed at building entrances to disinfect the skin. Frequent handwashing with soap and water or alcohol-based hand sanitizer, social distancing and practicing respiratory hygiene by covering the nose and mouth when sneezing or coughing remain WHO’s standard recommendations during this outbreak (11).

Finally, installing UV devices in public areas to disinfect objects and skin could instil a false sense of security in the public, thus reducing compliance with the WHO recommendations. Any use of UV irradiation for public disinfecting purposes should strictly follow the recommended guidelines for preventing unnecessary exposure to harmful UV rays.
